# Role of *TRIPTYCHON *in trichome patterning in *Arabidopsis*

**DOI:** 10.1186/1471-2229-11-130

**Published:** 2011-09-27

**Authors:** Martina Pesch, Martin Hülskamp

**Affiliations:** 1 Biocenter, Cologne University, Botanical Institute, Zülpicher Straße 47b, 50674 Cologne, Germany

## Abstract

**Background:**

Trichome patterning in *Arabidopsis thaliana *is governed by three types of activators, R2R3MYB, bHLH and WD40 proteins, and six R3MYB inhibitors. Among the inhibitors *TRIPTYCHON *(*TRY*) seems to fulfill a special function. Its corresponding mutants produce trichome clusters whereas all other inhibitors are involved in trichome density regulation.

**Results:**

To better understand the role of *TRY *in trichome patterning we analyzed its transcriptional regulation. A promoter analysis identified the relevant regulatory region for trichome patterning. This essential region contains a fragment required for a double negative feedback loop such that it mediates the repression of *TRY*/*CPC *auto-repression. By transforming single cells of *pTRY*:GUS lines with *p35S*:*GL1, p35S*:*GL3 *and *p35S*:*TTG1 *in the presence or absence of *p35S*:*TRY *or *p35S*:*CPC *we demonstrate that TRY and CPC can suppress the *TRY *expression without the transcriptional down regulation of the activators. We further show by promoter/CDS swapping experiments for the R3MYB inhibitors TRY and CPC that the TRY protein has specific properties relevant in the context of both, cluster formation and trichome density.

**Conclusions:**

Our identification of a *TRY *promoter fragment mediating a double negative feedback loop reveals new insight in the regulatory network of the trichome patterning machinery. In addition we show that the auto-repression by TRY can occur without a transcriptional down regulation of the activators, suggesting that the differential complex formation model has a biological significance. Finally we show that the unique role of TRY among the inhibitors is a property of the TRY protein.

## Background

Trichome patterning in *Arabidopsis thaliana *has become a well-studied model system to understand cell-cell communication in the context of two-dimensional pattern formation in plants [1-3]. Trichomes are formed in the basal part of young leaves [4]. The trichome position is not correlated with any recognizable leaf structures and clonal analysis excluded a cell lineage mechanism [5,6]. For these reasons, it is widely accepted that patterning is mediated by cellular interactions between initially equivalent cells [2,3,7].

Genetic screens have identified two classes of mutants governing this process. All patterning genes except for *TTG1 *have close homologs acting in a partially redundant manner [8-16]. The following summary will only consider the most relevant players as judged by the strength of the mutant phenotypes. One mutant class shows fewer or no trichomes. The corresponding genes are therefore considered positive regulators of trichome formation. The three most important positive regulators are the WD40 protein TRANSPARENT TESTA GLABRA1 (TTG1) [17-19], the R2R3 MYB related transcription factor GLABRA1 (GL1) [20], and the basic helix-loop-helix (bHLH)-like transcription factor GLABRA3 (GL3) [4,21,22].

In the second class, trichome clusters or a higher trichome density indicate a repressive role. The two most important inhibitors are the R3 single-repeat MYB factors TRIPTYCHON (TRY) and CAPRICE (CPC) [12,23]. Although, the two corresponding genes show high sequence similarity and an indistinguishable expression pattern in leaves [12], their mutant phenotypes suggest different modes of action. While the *cpc *mutant has a higher trichome density, the *try *mutant shows trichome clusters and a reduction in trichome number [4,12].

The expression pattern of most patterning genes is very similar. Initially, all genes are expressed ubiquitously in the cells at the leaf basis where trichome initials are formed (patterning zone). Later, expression increases in trichomes and disappears in epidermal cells [10-14,16,24-26]. The ubiquitous expression corresponds to the pre-pattern situation in which all cells are equivalent. During this phase the positive and negative regulators are considered to be engaged in regulatory feed-back loops that have several important features including the activation of the inhibitors by the activators, the repression of the activators by the inhibitors and the ability of the inhibitors to move between cells [14,15,27,28]. These create differences between the cells and ultimately result in a pattern of trichome and non-trichome cells [2,3].

After the initial pattern is established leaf growth leads to an increased spacing of trichomes without the formation of new trichomes. As in this phase patterning gene expression has ceased in epidermal pavement cells and increased in trichomes, the loss of trichome initiation competence is most likely due to the absence of activator gene expression. Whether activator gene expression in later leaf stages is generally shut off during leaf maturation or due to lateral repression by TRY and/or the other inhibitors is not clear.

The proposed regulatory feedback loop between the activators and the inhibitors ultimately leads to an auto-repression of the inhibitors. This could in principle be achieved in two ways. As the R3 single repeat MYB inhibitors lack a transcriptional activation region they could bind to promoter elements of the activators thereby preventing the transcription of the activators. As the repressors are activated by the activators the reduction of activator activity leads to reduced inhibitor transcription. Alternatively, the inhibitors could posttranslationally render the activation complex inactive [29]. Yeast two hybrid experiments showed that GL1 and TTG1 bind different regions of the GL3 protein suggesting that they form a trimeric transcriptional activation complex [22]. Binding of TRY or CPC to GL3 was shown to displace GL1 thereby inactivating the complex [14,29]. Although both mechanisms lead to a repression of the inhibitors they differ in their regulation scheme. The transcriptional repression of the activators by binding of TRY and/or CPC to the promoters would create a regulatory feed back loop that involves transcriptional down regulation of the activators. The postulated repression by differential complex formation would establish a shortcut of the regulatory feedback loop as the inhibitors can directly repress their own activation.

In this manuscript, we analyze the transcriptional regulation of *TRY *during trichome patterning. First, we determined the *TRY *promoter fragment relevant for *TRY *function and the specific basal and trichome-specific expression pattern. In addition we identified separate regions that are necessary for an enhancement of the specific expression pattern and showed that these regions are necessary for rescue. Second, we showed that TRY or CPC can repress the *TRY *expression directly without the transcriptional regulation of the activators by transforming single epidermal cells of *pTRY*:GUS lines with the three activators and TRY or CPC. Finally, we performed promoter swap experiments with *CPC *and *TRY *and tested the ability to rescue the *try *mutant trichome phenotypes. These experiments revealed specific properties of the TRY protein for clustering and trichome density regulation.

## Results

**Figure 1:**
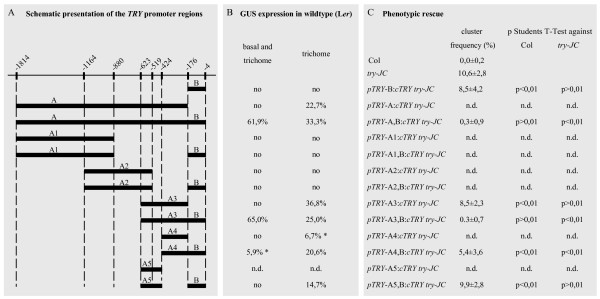
**A series of deletions in the 5' regulatory region of the *TRY *gene**. A) Schematic overview showing the relative positions of *TRY *promoter fragments with respect to the start codon of the different fragments. Each fragment is fused to the *CaMV 35S *minimal promoter and either to the GUS coding region or to the *TRY *CDS followed by the nopaline synthase terminator. Single fragments (A or B) or their fusion were used (A+B). B) Summary of the GUS expression data. We distinguish between the ubiquitous expression called "basal expression" and expression in trichomes. We found two categories, basal and trichome expression and expression only in trichomes. The percentage of analyzed independent T2 lines showing the respective expression category is provided. Data marked with a "*" showed exclusively weak staining as exemplified in Figure 2J. C) Overview of the rescue efficiency. It was determined by the ability to reduce trichome cluster formation in the *try-JC *mutant. The percentage of clusters relative to the number of trichome initiation sites was calculated on the first four leaves. Statistical difference for each rescue experiment in comparison to Col wild type or to the *try *mutant is determined through Student's t-test. The difference between the respective two means is significant for P < 0,01.

In a previous study, it was shown that a 4.2 kb genomic region containing a 1.8 kb 5' region and a 1.3 kb 3' region is sufficient to rescue the *try *mutant phenotype [12]. In a first step, we tested whether the 3' region or the introns are relevant for *TRY *function by transforming *try *mutant plants with a 1.8 kb 5' region that was fused to the *TRY *CDS (Figure 1, *pTRY*-A, B:c*TRY try-JC*). These plants showed complete rescue of the clustering phenotype indicating that the 1.8 kb 5' region contains all regulatory sequences necessary for the correct *TRY *expression in the leaf epidermis.

### Expression analysis of *TRY *promoter fragments

**Figure 2:**
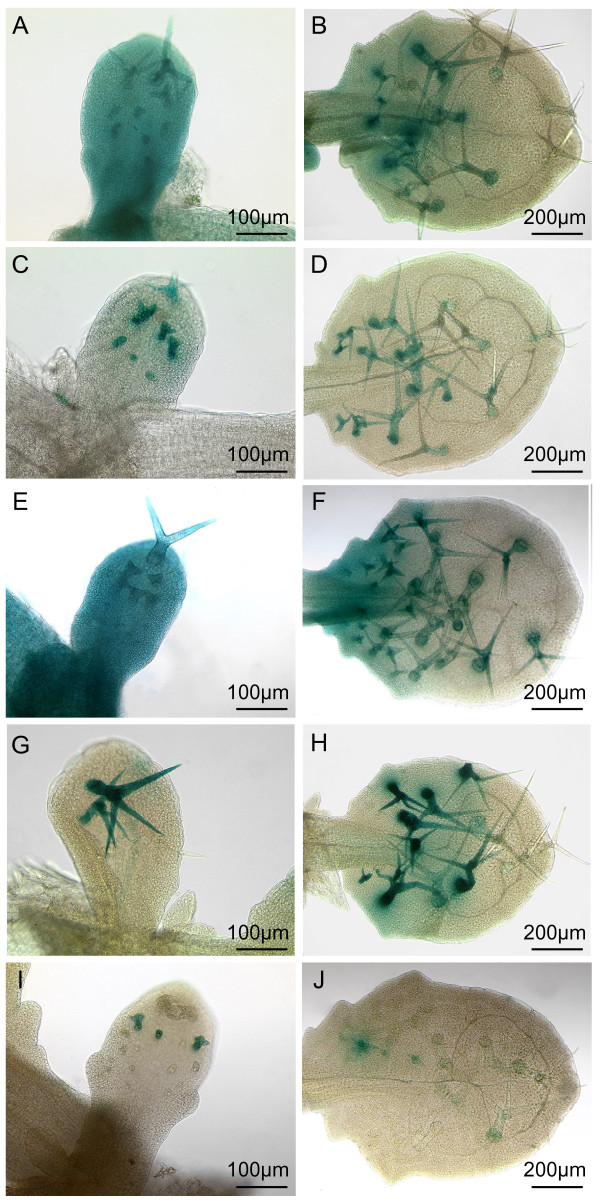
**Expression pattern of the 5' regulatory regions of the *TRY *promoter as revealed by GUS reporter gene expression**. The GUS expression pattern driven by different promoter fragments was monitored on young leaves at stages where new trichomes were still initiated (A, C, E, G and I) and for slightly older leaves in which trichome initiation had already stopped (B, D, F, H and J). Pictures were taken in each case from one of the strongest T2 lines carrying the respective *TRY *promoter GUS fusion construct: *pTRY*-A, B:GUS (A, B), *pTRY*-A:GUS (C, D), *pTRY*-A3, B:GUS (E, F), *pTRY*-A4, B:GUS (G, H) and *pTRY*-A5, B:GUS (I, J). Bars indicate the magnification of the images.

To identify specific regulatory elements, 5' promoter fragments were generated and their regulatory function monitored by fusion to the *p35S*-minimal promoter and the GUS marker gene (Figure 1A and 2). Because expression of a given construct is variable between different T2 lines we present pictures of the lines with the strongest expression only (Figure 2) and provide the percentage of lines in which the basal expression as well as trichome expression and those in which only the trichome specific expression is found after 24 hours of GUS staining (Figure 1B). Assuming that promoter elements driving a weak expression yield fewer transgenic lines with a strong expression this percentage is taken as an approximation of the expression strength of the promoter fragment under consideration.

For the expression analysis we initially used a fragment starting immediately upstream of an unique putative TATA Box located 32 base pairs upstream of the possible transcription start as determined by RACE PCR [12] (Figure 1A, *pTRY*-A). This fragment revealed GUS expression in trichomes, but the ubiquitous expression in young leaf regions (basal expression) observed before [12] was absent (Figure 2C, D). We therefore included the fragment immediately following the A-fragment and stretching to the -4 position relative to the ATG start codon (*pTRY*-B). *pTRY*-B represents the 5' UTR identified by Schellmann et al. and includes three possible transcriptional start sides suggested by ESTs (EH866228.1, AV533156.1, AI999616.1) and two putative TATA boxes (TATTA, TATAAA) [12,30-32]. A promoter fragment, *pTRY*-A, B, combining *pTRY*-A and *pTRY*-B revealed also trichome specific expression in 22,7% of the lines but in addition 61,9% of the lines (n = 35) showed the basal expression as well as the expression in trichomes. The *pTRY*-B fragment alone showed no expression. This indicates that the *pTRY*-B fragment is essential to enhance or stabilize the expression driven by the *pTRY*-A fragment.

A further deletion series revealed a minimal promoter region of about 620 bp (*pTRY*-A3, B). As found for the *pTRY*-A fragment the *pTRY*-B region is also necessary in the context of the *pTRY*-A3 fragment to mediate basal expression (Figure 2E, F). Further 5' deletion of about 200 bp (*pTRY*-A4, B) revealed trichome specific expression but only weak basal expression. Trichome-specific expression in these lines was only found in advanced stages of trichome development after branch formation (Figure 2G, H). The 200 bp fragment *pTRY*-A5, B revealed no basal expression and only sometimes a weak irregular expression in trichomes (Figure 2I, J).

These data do not allow to decide whether the *pTRY*-B fragment enhances both, the basal and trichome specific expression or whether it specifically regulates the basal expression. We therefore compared the expression pattern in *pTRY*-A3, B and *pTRY*-A3 at different time points of GUS staining procedure (data not shown). We observed that the GUS staining in young trichomes became detectable in both lines after two hours. While the basal expression in *pTRY*-A3, B became also detectable after two hours, no basal expression was detectable in *pTRY*-A3 even after 4 days of GUS staining. These data suggest, that the *pTRY*-B fragment specifically up-regulates the basal *TRY *expression in the context of the *pTRY*-A3 fragment.

### Identification of relevant promoter regions by rescue experiments

In order to test their functionality we used various promoter fragments to express the *TRY *CDS in *try-JC *mutant plants (Figure 1A, C). In order to avoid the problem that individual transformants may show a wide range of phenotypes we did not select individual lines for analysis in the T2 but directly analyzed the phenotype of T1 plants to hold account on the full phenotypic spectrum. In these experiments both, the *pTRY*-A, B and the *pTRY*-A3, B fragments fully rescued the clustering phenotype (Figure 1C). Expression of *TRY *driven by *pTRY*-A3, however, had no significant rescue ability (Figure 1C). The smaller fragment (*pTRY*-A4, B) only partially rescued the *try-JC *clustering phenotype. Together these data indicate that the *pTRY*-B fragment is essential for rescue.

### Regulation of the *TRY *promoter by TTG1, GL3, GL1 and TRY or CPC

In a next step we aimed to demonstrate the postulated activation/repression scheme of TTG1, GL3, GL1, TRY and CPC for the minimal *TRY *promoter fragment. The current models assume that TTG1, GL3 and GL1 can transcriptional activate the inhibitors *TRY *or *CPC *and that these in turn repress the activators and thereby also their own expression. The finding that TRY and CPC can compete with GL1 for binding to GL3 [14,29] suggests that the inhibitors can counteract the activity of the activators at the protein level directly. In this case TRY or CPC repress their own expression without a transcriptional repression of the activators.

**Figure 3:**
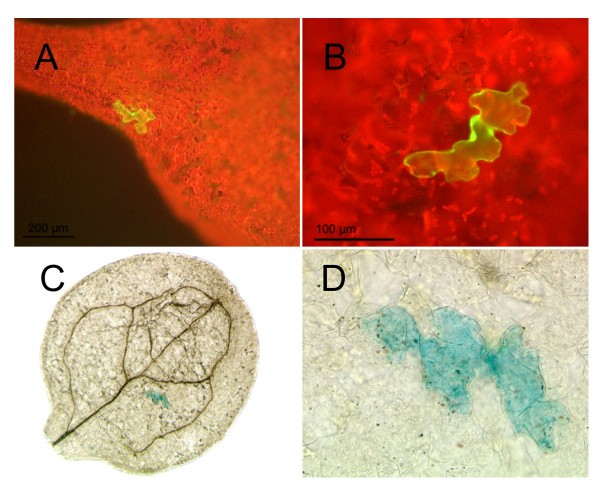
**Transient transformation of *pTRY*-A3, B:GUS cotyledons**. Single epidermal cells of *pTRY*-A3, B:GUS cotyledons were transiently transformed by particle bombardment. (A) Overview of a cotyledon with a single cell expressing the *p35S*:*GFP*:*YFP *control construct. (B) Higher magnification of a single cell expressing the *p35S*:*GFP*:*YFP *control construct. (C) Overview of a cotyledon with a single cell showing *pTRY*-A3, B:GUS expression after co-transformation with *p35S*:*GL1, p35S*:*GL3 *and *p35S*:*TTG1*. (D) Higher magnification of (C).

As *TRY *has been shown to be regulated by the activators in genetic experiments [27] the *TRY *promoter provides a tool to demonstrate that TRY/CPC can counteract the activators without a transcriptional repression of the activators. We used cotyledons for our analysis as no GUS expression was detected in this organ in *pTRY*-A3, B:GUS plants (Figure 3C). *GL1, GL3 *and *TTG1 *CDS under the control of the *p35S *promoter were used for transient transformations. In addition to these three constructs we added a *p35S*:*GFP*:*YFP *construct to control the bombardment efficiency. In four independent experiments analyzing each time 100 cells we found on average 68.2 ± 18.0% GUS expressing cells indicating that the simultaneous constitutive expression of *TTG1, GL3 *and *GL1 *induces the minimal *TRY *promoter fragment (Figure 3). Transformation with *35S*:*GFP*:*YFP *alone revealed no GUS positive cells.

In a second step we tested the model whether TRY or CPC can counteract the activity of the activators. Models derived from the finding that TRY and CPC compete with GL1 for binding to GL3 in yeast three hybrid experiments suggest that differential complex formation renders the proposed activator complex inactive [14,29]. We took advantage of the fact that in this experimental setup any indirect repression of *TRY *by TRY or CPC through the transcriptional repression of the activators is excluded as their expression is under the control of the *35S *promoter. In four independent experiments with 100 cells in each experiment we found only 0.2 ± 0.5% or 0.2 ± 0.4% GUS-positive cells when expressing *p35S*:*TRY *or *p35S*:*CPC *respectively in addition to the three activators. This indicates that TRY and CPC can counteract *TRY *activation by the three activators without a transcriptional repression of the activators.

### Relevance of MYB and MYC binding sites

**Figure 4:**
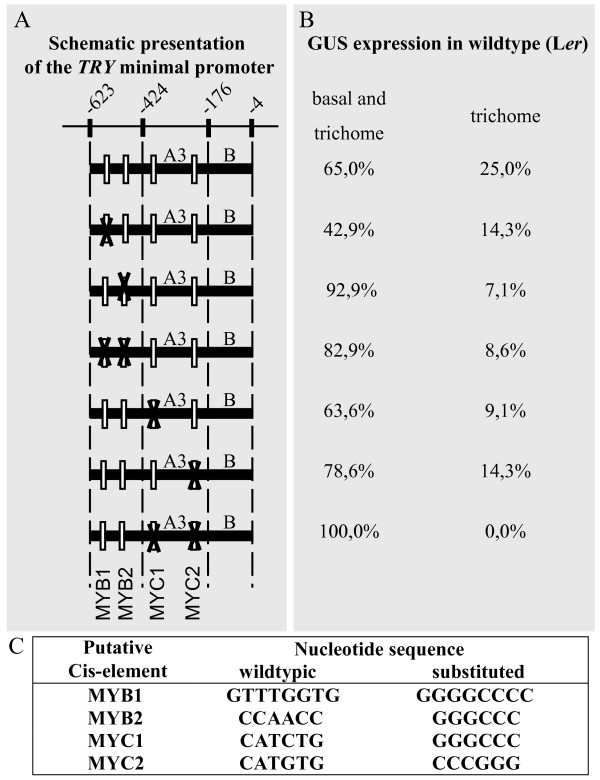
**The minimal 5' regulatory region of the *TRY *gene including the putative analyzed MYB and MYC binding sites and their substitutions**. A) Schematic overview showing the relative position of the minimal promoter with respect to the start codon. Each fragment is fused to the CaMV *35S *minimal promoter and to the GUS coding region followed by the nopaline synthase terminator. The white boxes symbolize the relative position of the analyzed MYB and MYC binding sites. The black crosses shows which binding site is mutated in the respective fragment. B) Summary of the GUS expression data. We distinguish between the ubiquitous expression called "basal expression" and expression in trichomes. We found two categories, basal and trichome expression and expression only in trichomes. The percentage of analyzed independent T2 lines showing the respective expression category is provided. C) List of the analyzed binding sites with their corresponding wild type nucleotide sequence and the sequence used for base substitution.

Our finding that the activators can activate the *TRY *promoter in transient expression assays together with the finding that GL3 and GL1 bind to the *TRY *promoter in ChIP experiments [33,34] prompted us to search for putative MYB and MYC (bHLH factor) binding sites. We found five putative MYB and two putative MYC sites in the *pTRY*-A3, B fragment that was the minimal promoter fragment for full rescue of the *try *phenotype (Table 1). Among the five putative MYB sites two seemed most promising as they were identified in the context of regulatory pathways in other plants that are also regulated by TTG1-dependent pathways [35]. In addition the MYB factor binding to these MYB binding sites are in the same clade in the phylogenetic tree as GL1 [36]. We therefore focused on these two putative binding sites. To determine the role of the two selected MYB and the two MYC sites in the regulation of the correct expression pattern we mutated each site individually and both MYB and both MYC sites together (Figure 4C). None of the mutated constructs showed a marked reduction or even absence of *pTRY-*A3, B:GUS expression (Figure 4B). However, we noted differences such that the MYB1 site has the most positive effect on the basal expression whereas the MYB2 and the MYC sites have a repressive role. As a *pTRY-*A3, B:TRY construct containing mutations in both MYC sites resulted in a complete rescue of the *try *mutant clustering phenotype these sites do not appear to be relevant in this context (Additional File 1)

**Table 1:** Overview of the identified MYB and MYC binding sites in the 5'-TRY minimal promoter identified by PLACE database

	5'-*TRY*- nucleotide sequence	Position relative to the ATG (start/end)	Name of the described cis-element	Putative cis-element nucleotide sequence	Description of the putative cis-element
**MYB1**	GTTTGGTG	-544/-551	MYBPLANT	MACCWAMC	Binding of AmMYB305 in *Antirrhinum majus *to box P from gPAL2 of *Phaseolus vulgaris; *P box related sequences [48,49] are identified in several promoters of phenylpropanoid biosynthesis related genes (PAL, CHS, CHI, DFR, BZ1) in different plants (*Phaseolus vulgaris, Antirrhinum majus, Petunia hybrid*a, *Petroselinum crispum, Arabidopsis thaliana, Zea mays*) [50]
**MYB2**	CCAACC	-531/-536	MYBPZM	CCWACC	Binding in promoters of A1 and BZ1 genes of phlobaphene pigmentation and flavonoid biosynthesis in *Zea mays *(factors, e.g. C1, P) [51]
**MYB3**	TTTGTTA	-607/-613	MYBGAHV	TAACAAA	Central element of the gibberellin (GA) response complex (GARC) in the high-pI alpha-amylase gene in *Hordeum vulgare*, binding of GaMYB [52-54].
**MYB4**	CCGTT	-153/-157	MYBCOREATCYCB1	AACGG	"Myb core" found in the promoter of *Arabidopsis thaliana *cyclin B1:1 gene [55].
	GCCGTTCGT	-150/-158	v-MYB*	NSYAACGGN	Binding site of the v-MYB oncocgene of the avian myeloblastosis virus [56].
	GGCCGTTCGT	-150/-159	c-MYB*	NNNAACKGNC	Binding site of the c-MYB, the cellular homolog of v-MYB
**MYB5**	TTGAACTTGC	-404/-413	c-MYB*	NNNAACKGNC	Binding site of the c-MYB, the cellular homolog of v-MYB
**MYC1**	CATCTG	-399/-404	MYC CONSENSUSAT	CANNTG	Binding of AtMYC2 in pAtRD22 (dehydration responsive gene) in *Arabidopsis thaliana*.
**MYC2**	CATGTG	-243/-248	MYC CONSENSUSAT	CANNTG	Binding of AtMYC2 in pAtRD22 (dehydration responsive gene) in *Arabidopsis thaliana *[48,49].
			MYCATERD1	CATGTG	Binding of AtNAC to the ERD1 gene (early responsive to dehydration) in dehydrated *Arabidopsis thaliana *[57,58].
			MYCATRD22	CACATG	Binding of AtMYC2 to the RD22 gene (dehydration responsive gene) ) in *Arabidopsis thaliana *[49].

An additional analysis marked by "*" was done by the TRANSFAC database.

### The *pTRY*-B region mediates the repression of the inhibitors repression

**Figure 5:**
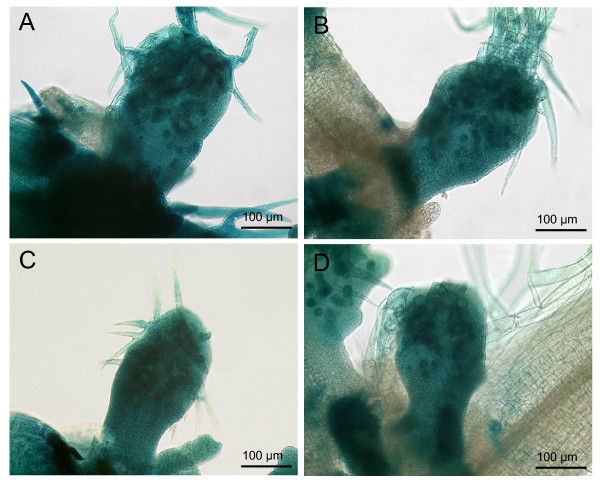
**Expression pattern of 5' regulatory regions of the *TRY *promoter in the *cpc try *double mutant as revealed by GUS reporter gene expression**. The GUS expression pattern driven by different promoter fragments was monitored on young leaves at stages when new trichomes are still initiated. Pictures were taken in each case from one of the strongest T2 lines carrying the respective *TRY *promoter GUS fusion construct in the *cpc-1 try82 *double mutant: (A) *pTRY*-A, B:GUS, (B) *pTRY*-A3, B:GUS, (C) *pTRY*-A3:GUS, (D) *pTRY*-A4, B:GUS.

In a separate line of experiments we tested, whether reduced *pTRY*:GUS expression in the absence of the *pTRY*-B fragment or in the *pTRY*-A4, B lines is caused by endogenous R3MYB repressor activity. We compared the expression of the *pTRY*-A3, B, *pTRY*-A3 and *pTRY*-A4, B constructs in wild type and the *cpc try *mutant background (Figure 5). All constructs revealed a strong basal and trichome specific GUS expression. Thus the lack of basal expression in the *pTRY*-A3 line and the lack of basal and most of the trichome specific expression in the *pTRY*-A4, B line is rescued in the *cpc try *mutant. These data suggest that the -424 to -176 fragment (*pTRY*-A4) promotes the basal expression and that the *pTRY*-B fragment and the -623 to -424 (*pTRY*-A5) fragments mediate repression of the inhibitors repression.

In order to show that this repression of the inhibitors repression involves the patterning activators we used transient expression assays. The *p35S*:*GL1, p35S*:*GL3 *and *p35S*:*TTG1 *constructs were co-bombarded in wild type and *cpc try *mutants carrying the *pTRY*-A3, B, *pTRY*-A3 and *pTRY*-A4, B constructs (Table 2). In wild type we found GUS-positive cells only in plants carrying the *pTRY*-A3, B construct (n = 100). By contrast, all constructs revealed GUS-positive cells in the *cpc try *mutant background indicating that this regulation event involves the trichome patterning activators. Furthermore the percentage of GUS-positive cells per transformed epidermal cells was much higher for *pTRY*-A3, B transformed cotyledons in *cpc try *double mutant indicating a stronger activation.

**Table 2:** Co-transformation promoter activation assay in *Arabidopsis *cotyledons

	Ler*	cpc-1 try-82*
*pTRY*-A3, B	53.7 ± 4.4	118.8 ± 18.2
*pTRY*-A3	0.5 ± 0.6	80.2 ± 8.2
*pTRY*-A4, B	0.0 ± 0.0	32.7 ± 3.0

* Percentage [%] of GUS activated epidermal cotyledon cells after overnight staining relative to the number of GFP:YFP marked epidermal cotyledon cells from four independent experiments (each experiment included 100 cells).

### Specific properties of the TRY protein in the regulation of cluster formation and trichome density

**Figure 6:**
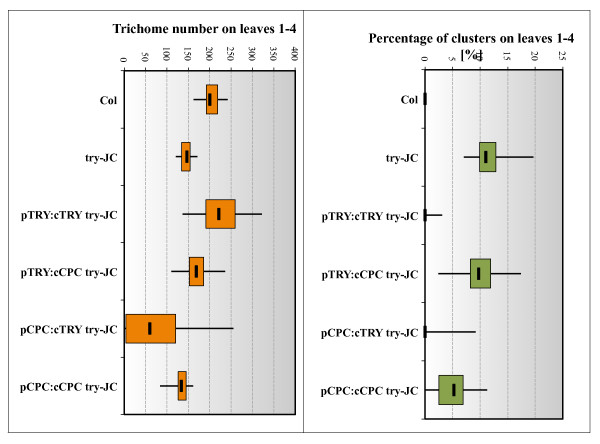
**A box-Whisker-plot of the trichome number and cluster frequency of the *TRY*/*CPC*- promoter swap experiments**. Monitored are *try-JC *(n = 30), Col (n = 30), and *pTRY:cTRY, pTRY:cCPC, pCPC:cTRY, pCPC:cCPC *in *try-JC *mutant background (each n = 50 T1 plants). As coding sequence the CDS (c) of either *TRY *or *CPC *and the promoter *pTRY*-A3, B and *pCPC *(-686 to -158) were used. (A) The trichome number is summed for the first four leaves. (B) The percentage of the cluster is calculated relative to the initiation sites for the first four leaves. The boxes themselves contain the middle 50% of the data. The upper line of the box marks the 75th percentile and the lower one the 25th percentile. The line in the box indicates the median value. The ends of the vertical lines indicate the minimum and maximum data values.

The fact that among the six R3 single repeat MYB inhibitor genes only mutations in *TRY *lead to a clustering phenotype raised the question, whether the transcriptional regulation of *TRY *or its protein properties constitute this difference. We therefore compared reciprocal swaps of promoters and CDS of the *TRY *and *CPC *genes for their ability to rescue the *try *mutant. *CPC *was chosen because it represents the main inhibitor for trichome density regulation and because it has a similar expression pattern including the early ubiquitous and later trichome specific expression. Here we chose 525 bp of the 5' upstream region of the *CPC *gene, which showed the expected *CPC *expression in leaves and roots (Additional File 2), and was able to rescue the *cpc *mutant trichome phenotype when fused to the *CPC *CDS (Additional File 3). Both combinations containing the CDS of *TRY, pTRY*:*cTRY *and *pCPC*:*cTRY*, completely rescued the clustering phenotype (Figure 6). By contrast, the combination of the *TRY *promoter with the CDS of *CPC *exhibited no significant rescue (Figure 6). This indicates that the specific role of *TRY *in preventing cluster formation is not based on its transcriptional regulation but on specific protein properties.

We also determined the trichome number in these rescued lines. In this respect *try *mutants have the opposite effect as all the other inhibitor mutants in showing fewer trichomes than wild type [12]. Expression of *TRY *under the control of the *TRY *promoter can significantly rescue the trichome number. By contrast, the *pTRY*:*cCPC *construct revealed weak but not significant rescue. When using the *CPC *promoter we found no rescue with the *pCPC*:*cCPC *construct and an overexpression phenotype leading to less or even no trichomes in *pCPC:cTRY *plants. Thus in summary, we recognized protein-specific properties of TRY in the context of *TRY *dependent trichome density regulation. The finding that the *TRY *CDS driven by the *CPC *promoter could not rescue the *try *density defect suggests additional relevant differences between the two promoters in this context.

## Discussion

In this study we analyzed the transcriptional regulation of *TRY *to learn more about the unique role of *TRY *in trichome patterning among the R3MYBs homologs as judged by the clustering phenotype of the *try *mutants.

### Role of the *pTRY*-B fragment: general enhancer/suppressor or regulator of basal expression

Our promoter analyses revealed an important role of the *pTRY*-B fragment as it is absolutely necessary for the basal expression in the young leaf and for rescuing the clustering phenotype of the *try *mutant. It seems to modulate the spatial-temporal expression pattern. Several findings suggest that *pTRY*-B is specifically required for the basal expression of *TRY*. First, we never found any basal expression in the absence of the *pTRY*-B fragment in wild type background. Second, the expression in trichomes is similarly strong (as judged by the GUS staining time course experiments) with and without the *pTRY*-B fragment and the basal expression co-appears with the trichome specific expression in the presence of the *pTRY*-B fragment, but is not seen after 4 days without.

The absolute requirement of *pTRY*-B for rescue of the *try *clustering phenotype immediately suggests that the basal expression of *TRY *is relevant for patterning. This finding would be an important piece of support for the current theoretical models [2]. As the pattern is generated in a field of initially equivalent cells, the patterning system needs to start with an initially ubiquitous expression of activators and inhibitors that is necessary for the establishment of a pattern. Thus, according to this scenario the requirement of the *pTRY-B *fragment and therefore also of the basal expression supports this type of model.

How is the basal *TRY *expression regulated by the *pTRY*-B fragment? An answer towards this end comes from our analysis of the *pTRY*-A3, B:GUS and *pTRY*-A3:GUS constructs in *try cpc *mutants. The findings that the *pTRY*-A3 fragment can be activated by GL1 GL3 and TTG1 in the *try cpc *double mutants but not in wild type together with the presence of basal expression in *pTRY*-A3 *try cpc *plants indicates that the *pTRY*-B fragment mediates the repression of the *TRY *repression by TRY or CPC. Thus the apparent requirement of the *pTRY*-B fragment for basal expression is in fact a double negative regulatory event. The current data suggest that the -424 to -176 fragment is important for turning on the basal expression and that TRY/CPC inhibit this activation with the immediate upstream -623 to -424 and downstream -176 to -4 regions counteracting this inhibition. As the absence of the MYB2 site leads to an increased basal expression it is possible that this site is involved in this regulation loop. Similarly, the higher basal expression upon the deletion of the two MYC sites can be interpreted as a function of these sites in the double negative repression loop.

### Self-repression of *TRY *without transcriptional regulating of the activators

Models explaining trichome formation in *Arabidopsis *are derived from the activator-inhibitor model formulated by Meinhardt und Gierer [37]. This theoretical model explains pattern formation with two components: an activator activates its own inhibitors and its own expression with the inhibitor being able to move faster than the activator.

When adapting this theoretical model to the biological context there are two possibilities. First, the inhibitor down-regulates its own expression indirectly through the down regulation of the activator. Second, the inhibitor represses its own expression through competitive complex formation [27,29,38]. While we can not exclude the first possibility, our data show that the second scenario is sufficient. We show that the minimal *TRY *promoter can be ectopically activated by the combined expression of GL1, GL3 and TTG1 in cotyledon cells. As the three activators are expressed under the control of the *35S *promoter any transcriptional feed back loops involving these three genes are unlikely to be relevant in this experiment. The repression of the activity of the three activators by TRY or CPC provides evidence that TRY and CPC represses the *TRY *expression directly rather than through a transcriptional feed back loop involving the activator genes.

### Specific properties of TRY protein for patterning

To further understand the molecular nature of the uniqueness of *TRY *among the six R3-single repeat MYB inhibitors, we used promoter swap experiments with *CPC *which shares all aspects of the *TRY *expression pattern as judged by promoter:GUS analysis. This enabled us to study the relevance of the transcriptional regulation and protein function of both genes in the *try *mutant cluster formation and density phenotypes. We found a different behavior of TRY and CPC proteins in these rescuing experiments such that only TRY protein could rescue the *try *mutant clustering and density phenotype when expressed under the *TRY *promoter. A similar situation was found in *cpc *mutant rescue experiments where the TRY protein expression under the control of the *CPC *promoter resulted in a stronger overexpression phenotype as compared to the CPC protein [39]. A contribution of *TRY *specific promoter properties was only found in the context of trichome density regulation. This is contrast to the behavior of *ETC3 *another homolog of *TRY *and *CPC*. The *etc3 *mutant could be rescued by ETC3 in the same manner by regulation through the *ETC3, CPC *and *TRY *promoter, so that the promoters were interchangeable with respect to the trichome density phenotype in the *etc3 *mutant but not with respect to the *try *mutant [14]. Thus TRY dependent regulation of trichome density is dependent on both, specific protein properties as well as specific aspects of transcriptional regulation.

The observed differences between TRY and CPC protein functions could in principle be due to various aspects including the protein stability, protein movement and their interaction with other proteins, in particular the bHLH factors. Both proteins have been shown to interact with bHLH factors [10,11,13,14,16,40]. Their interactions, however, seem to differ as CPC binds stronger to GL3 [41] and suppresses the binding of GL1 to GL3 more efficiently than TRY [14]. Different strength in their binding to GL3 is also likely to change the intercellular movement of TRY and CPC [14]. Both proteins have been shown to move between cells and share a 79 bp N-terminal region in which W76 and M78 were shown to be necessary for movement of CPC and are conserved in the TRY protein [27,28,38]. However, TRY protein is lacking the first 9aa that were also be shown to be necessary for CPC movement [28] and could therefore in principle be responsible for a different movement behavior. The most obvious difference between the TRY protein and CPC, ETC1, ETC2, ETC3 and TCL1 is its c-terminal extension of unknown function [41]. While we begin to understand the functional diversification of the R3 single MYB factors in trichome development it is still elusive which properties are responsible for the differences in their requirement for clustering and density control.

## Conclusions

In this work we show that the auto-repressive effect of TRY does not require a transcriptional downregulation of the activators suggesting that differential complex formation is biologically relevant. We further show that the unique role of TRY among the inhibitors is a property of the TRY protein. Finally our analysis of the *TRY *promoter lead to the identification of a 620 bp fragment sufficient to rescue the *try *mutant phenotype. It contains a fragment that mediates the repression of its own repression suggesting a complex regulation scheme. It is likely, that we are seeing here just the tip of an iceberg, as the transcriptional regulation of *TRY *has additional complexity at the level of organ specificity involving additional regulatory genes such as the well studied *SQUAMOSA PROMOTER BINDING PROTEIN LIKE ***(***SPL*) gene [42].

## Methods

### Plant lines and growth conditions

Plants were grown on soil at 24°C in a 16 h light/8 h dark cycle. Plant transformations were performed by the floral dip method [43]. The transgenic *pTRY*-A3, B:GUS L*er *and *cpc-1 try-82 *double mutant [12] line was generated by a genetic cross. Complementation experiments were done in *try-JC *and *cpc-1 *mutants [23,44] and Col-0 and WS-0 respectively as a control. For the transient transformation of cotyledons *pTRY*-A3, B:GUS, *pTRY*-A3:GUS and *pTRY*-A4, B:GUS lines in L*er *or in *cpc-1 try-82 *double mutant background were used. For these experiments surface sterilized seeds were grown on MS plates containing 1% sucrose and 20 μg/ml Basta for 7 days at 22°C with 16 h light/8 h dark cycle.

#### Constructs

##### Construction of the *TRY *and *CPC *promoter fragments

L*er pTRY-B *was cloned as a HindIII fragment in pGEM-T-easy. All other promoter fragments were cloned in pDONR201 by BP reactions (Invitrogen). Promoter mutations were introduced by PCR based site directed mutagenesis (details are available on request). pENTR1A-w/o-ccdB was created by deleting the EcoRI fragment to take out the Gateway recombination cassette inside the attB sequences of pENTR1A. All fragments were verified by sequencing. (Detailed primer information see Additional File 4).

##### *CaMV 35S *minimal promoter GUS and CDS constructs

The basic Gateway destination vector PARB (pANGUS-Gateway RekombinationscasetteA-Basta-resistence) was created in several steps. The *CaMV 35S *minimal promoter (-46 to +7) fused to the GUS gene from pBT-GUS [45] was cloned as a BamHI and XmaI fragment into pPAM (GenBank AY027531). The Gateway recombination cassette A (Invitrogen) was cloned as a BcuI and SalI fragment in pANGUS (pANGUS-RecA). The kanamycin resistance was replaced by the bar gene with nos-promoter and nos-terminator from pGREEN-Bar as a RsrII and SpeI fragment. The *pTRY*-B fragment was cloned into the HindIII site of PARB directly in front of the *35S *minimal promoter to create PARB-B. PARB-*TRY*-CDS, PARB-B-*TRY*-CDS, PARB-*CPC*-CDS are derivates of PARB and PARB-B in which the GUS gene was replaced by the *TRY *or *CPC *CDS (L*er*).

Promoter-GUS- and promoter-CDS constructs were generated by LR recombinations (Invitrogen) using the entry clones *pCPC*-pDONR201, pENTR1A-w/o-ccdB and the different entry clones of the deletion and substitution series of *pTRY *and the different destination vectors derived from PARB.

##### Effector constructs for transient co-transformation experiments of *Arabidopsis *epidermal cotyledon cells

CDS's from L*er *were cloned in pENTR1A or by BP recombination in pDONR201. The effector constructs (*p35S*:*GL1, p35S*:*GL3, p35S*:*TTG1, p35S*:*TRY, p35S*:*CPC*) and the control *p35S*:*GFP*:*YFP *were created by LR recombination of the respective entry clones with pAMPAT-GW.

#### Histochemical analysis and microscopy

GUS activity was assayed as described previously [46]. For light microscopy we used a Leica DMRE microscope. Images were taken with a KY-F70 3-CCD JVC camera and DISKUS software (DISKUS, Technisches Büro). In all experiments 35 independent T2 lines were used for statistical analysis.

#### Evaluation of the trichome initiation sites and cluster frequency

Trichome initiation sites and the number of trichome clusters were counted on the first four fully expanded leaves on 50 individual T1 plants after Basta selection and on 30 Col-0, WS-0, *try-JC *and *cpc-1 *plants. The significance of the difference between complemented plants and either Col or *try-JC *was tested by Student's T-test (two-tailed distribution and two-sample equal variance, P < 0,01). Trichome density and cluster frequency data in the promoter CDS swap experiment are shown as box-whisker-plots. The quartile function of Excel (Microsoft Office Standard 2007) was used to return the five quartiles for the data sets (minimum value, first quartile (25th percentile), median value, third quartile (75th percentile) and maximum value). The plot itself was created with a free accessible boxplot template (http://www.austromath.at/medienvielfalt/materialien/beschreibendeStatistik/content).

#### Microprojectile Bombardment

Transient *TRY *expression analysis was carried out by using the particle bombardment method in *Arabidopsis *cotyledons [47]. Each set of experiment was done independently at least four times. After bombardment plants were grown for 24 h and the number of transformed cells was determined by the presence of the co-bombarded *p35S*:*GFP*:*YFP*. After overnight GUS staining and tissue clearing the number of GUS stained cells was determined and the percentage of GUS positive cells relative to the transformed cells calculated.

### *In silico *analysis of the *TRY *promoter

To identify transcription factor binding sites the Plant Cis-acting Regulatory DNA Elements (PLACE, http://www.dna.affrc.go.jp/PLACE/) and the TRANSFAC (TFSEARCH: Searching Transcription Factor Binding Sites (ver 1.3), http://mbs.cbrc.jp/papia/) databases were used.

## Abbreviations

TRY: Triptychon; CPC: Caprice; GFP: Green fluorescent protein; GL1: Glabra1; GL3: Glabra3; TTG1: Transparent testa glabra1; YFP: Yellow fluorescent protein; GUS: Glucoronidase; ETC3: Enhancer of triptychon and caprice3; TCL1: Trichomless1; SPL: Squamosa promoter binding protein like (SPL)

## Authors' contributions

MP carried out all molecular and genetic studies. MH participated in the design and coordination of the work and wrote the manuscript. All authors have read and approved the final manuscript.

## Supplementary Material

Additional file 1 **Functional relevance of MYC1 and MYC2 sites**. A box-Whisker-plot of the trichome number and cluster frequency of the double MYC binding site mutated *TRY *promoter rescue experiment. For *pTRY-A3, B:cTRY *and *pTRY-A3, B-mutMYC+mutMYC2:cTRY *in *try-JC *mutant background 50 T1 plants are monitored and 30 plants for Col and *try-JC*. The boxes contain the middle 50% of the data. The upper line of the box marks the 75th percentile and the lower one the 25th percentile. The line in the box indicates the median value. The ends of the vertical lines indicate the minimum and maximum data values. Click here for file

Additional file 2 **Expression analysis of the CPC promoter**. GUS expression of the 5' regulatory region of the *CPC *promoter. GUS staining was observed for a young leaf executing trichome patterning (A) or a young leaf already finished trichome patterning (B). In addition a 7 days old primary root grown on MS medium was shown (C). Pictures were taken from one T2 line representative for 35 independent observed lines. Bars as indicated. Click here for file

Additional file 3 **Trichome rescue by *pCPC*:*cCPC***. A box-Whisker-plot of the trichome number and cluster frequency of the *pCPC:cCPC *rescue experiment. Monitored are WS-0 (n = 30), *cpc-1 *(n = 30), and *pCPC*:*cCPC *in *cpc-1 *mutant background (n = 50 T1 plants). The CDS of *CPC *was expressed under the control of *pCPC *(-686 to -158). The boxes contain the middle 50% of the data. The upper line of the box marks the 75th percentile and the lower one the 25th percentile. The line in the box indicates the median value. The ends of the vertical lines indicate the minimum and maximum data values. Click here for file

Additional file 4 **Primer list**. The table shows a list of the relevant primers used for the creation of the constructs. Click here for file
