# Supplementary figures and images for: Role of TRIPTYCHON in trichome patterning in Arabidopsis
Source: BMC Plant Biol. 2011 Sep 27;11:130. doi: 10.1186/1471-2229-11-130 (PMC3196707; doi:10.1186/1471-2229-11-130)

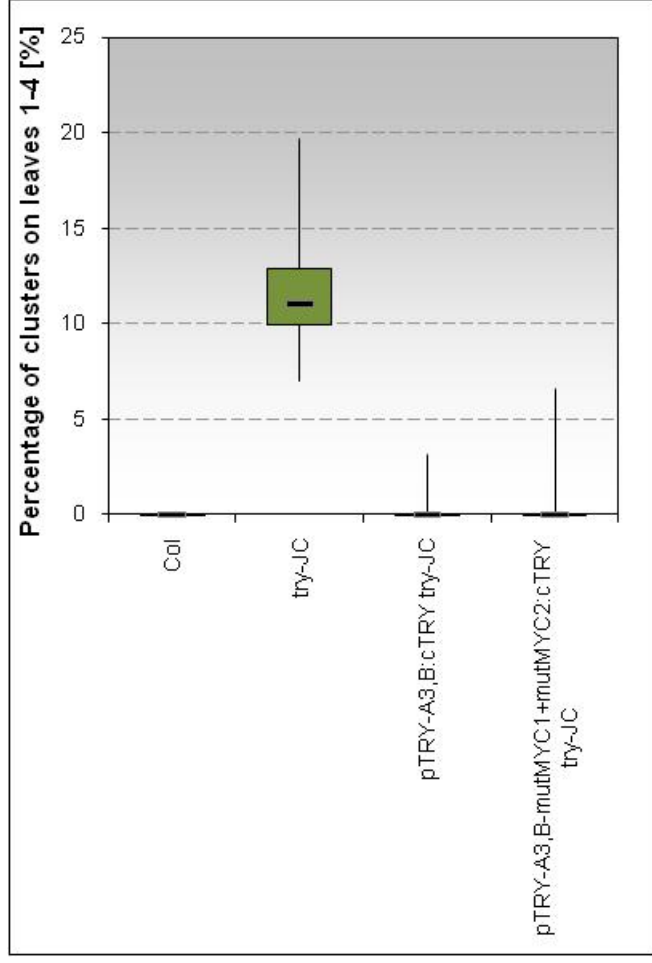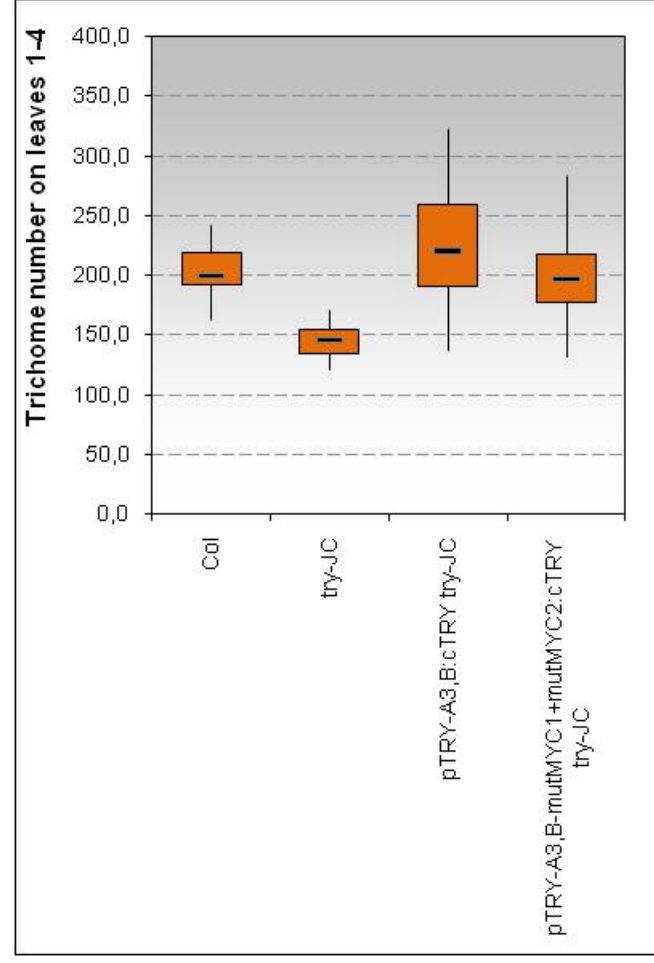

Supplement: Additional file 1 — Functional relevance of MYC1 and MYC2 sites. A box-Whisker-plot of the trichome number and cluster frequency of the double MYC binding site mutated TRY promoter rescue experiment. For pTRY-A3, B:cTRY and pTRY-A3, B-mutMYC+mutMYC2:cTRY in try-JC mutant background 50 T1 plants are monitored and 30 plants for Col and try-JC. The boxes contain the middle 50% of the data. The upper line of the box marks the 75th percentile and the lower one the 25th percentile. The line in the box indicates the median value. The ends of the vertical lines indicate the minimum and maximum data values. [file 1471-2229-11-130-S1.PDF]

**A****100  $\mu$ m**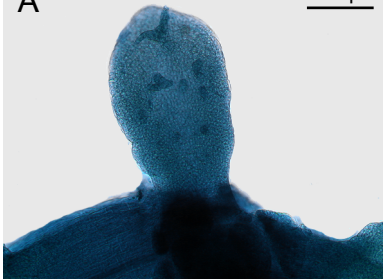**B****200  $\mu$ m**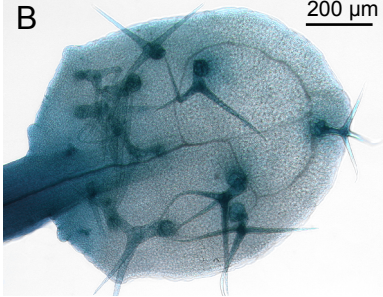**C****100  $\mu$ m**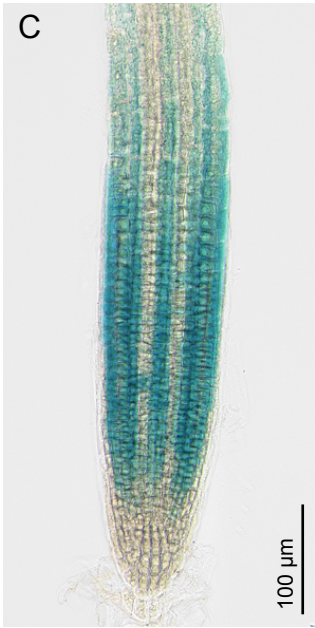

Supplement: Additional file 2 — Expression analysis of the CPC promoter. GUS expression of the 5' regulatory region of the CPC promoter. GUS staining was observed for a young leaf executing trichome patterning (A) or a young leaf already finished trichome patterning (B). In addition a 7 days old primary root grown on MS medium was shown (C). Pictures were taken from one T2 line representative for 35 independent observed lines. Bars as indicated. [file 1471-2229-11-130-S2.PDF]

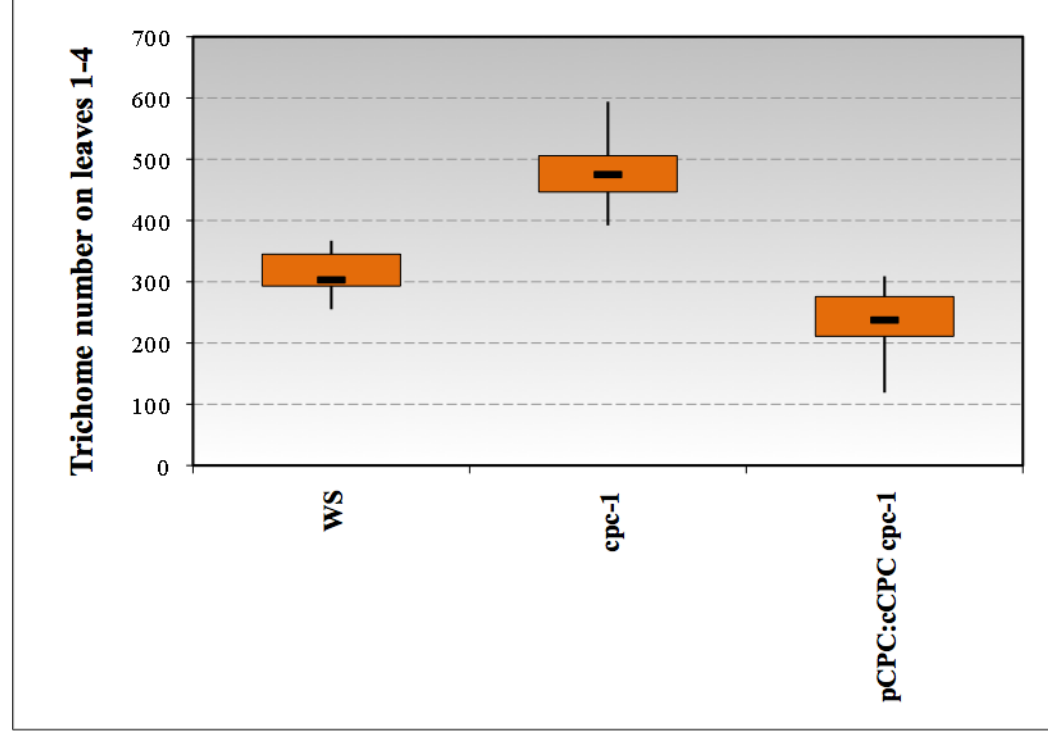

Supplement: Additional file 3 — Trichome rescue by pCPC:cCPC. A box-Whisker-plot of the trichome number and cluster frequency of the pCPC:cCPC rescue experiment. Monitored are WS-0 (n = 30), cpc-1 (n = 30), and pCPC:cCPC in cpc-1 mutant background (n = 50 T1 plants). The CDS of CPC was expressed under the control of pCPC (-686 to -158). The boxes contain the middle 50% of the data. The upper line of the box marks the 75th percentile and the lower one the 25th percentile. The line in the box indicates the median value. The ends of the vertical lines indicate the minimum and maximum data values. [file 1471-2229-11-130-S3.PDF]
