# Supplementary material for: Role of TRIPTYCHON in trichome patterning in Arabidopsis
Source: BMC Plant Biol. 2011 Sep 27;11:130. doi: 10.1186/1471-2229-11-130 (PMC3196707; doi:10.1186/1471-2229-11-130)
Supplement: Additional file 4 — Primer list. The table shows a list of the relevant primers used for the creation of the constructs. [file 1471-2229-11-130-S4.DOC]

**Additional File 1**

| | **pTRY-Promoter Fragments** | **Primer Name** | Primer Sequence | | --- | --- | --- | | A | TRY-F1-sen-attB1 | GGGGACAAGTTTGTACAAAAAAGCAGGCTTAAAGAGATATTTTACCAACTAGGC | |  | TRY-F3-asen-attB2 | GGGGACCACTTTGTACAAGAAAGCTGGGTTTTTTTTTTCAGGGTTGGAC | | A1 | TRY-F1-sen-attB1 | GGGGACAAGTTTGTACAAAAAAGCAGGCTTAAAGAGATATTTTACCAACTAGGC | |  | TRY-F1-asen-attB2 | GGGGACCACTTTGTACAAGAAAGCTGGGTATGGGAATAATATTTATATC | | A2 | TRY-F2-sen-attB1 | GGGGACAAGTTTGTACAAAAAAGCAGGCTGAAAGAAAAGTCAATCGGGAATGAG | |  | TRY-F2-asen-attB2 | GGGGACCACTTTGTACAAGAAAGCTGGGTTTTTTTTTTCAGGGTTGGAC | | A3 | TRY-F3-sen-attB1 | GGGGACAAGTTTGTACAAAAAAGCAGGCTGAAAGAAAAGTCAATCGGGAATGAG | |  | TRY-F3-asen-attB2 | GGGGACCACTTTGTACAAGAAAGCTGGGTTTTTTTTTTCAGGGTTGGAC | | A4 | TRY-F4-sen-attB1 | GGGGACAAGTTTGTACAAAAAAGCAGGCTCTAAAACATCGTTGAACTTG | |  | TRY-F3-asen-attB2 | GGGGACCACTTTGTACAAGAAAGCTGGGTTTTTTTTTTCAGGGTTGGAC | | A5 | TRY-F3-sen-attB1 | GGGGACAAGTTTGTACAAAAAAGCAGGCTGAAAGAAAAGTCAATCGGGAATGAG | |  | MP-TRY-ohne MYC1-attB2 | GGGGACCACTTTGTACAAGAAAGCTGGGTGTTATTGATTTGTTGTTGTTC | | B | 5´-TRY-179-HindIII-for | CAAGCTTACTATTAGTTTTGCAAAGGCCGTT | |  | 5´-TRY-179-HindIII-rev | CAAGCTTTATTGAAGTAAGAAAAGAAAAATAGAGAG | | A3,B | TRY-F3-sen-attB1 | GGGGACAAGTTTGTACAAAAAAGCAGGCTGAAAGAAAAGTCAATCGGGAATGAG | |  | 5´-TRY-179-rev-attB2 | GGGGACCACTTTGTACAAGAAAGCTGGGTCAAGCTTTATTGAAGTAAGAAAAGAAAAATAGAGAG | | A4,B | TRY-F4-sen-attB1 | GGGGACAAGTTTGTACAAAAAAGCAGGCTCTAAAACATCGTTGAACTTG | |  | 5´-TRY-179-rev-attB2 | GGGGACCACTTTGTACAAGAAAGCTGGGTCAAGCTTTATTGAAGTAAGAAAAGAAAAATAGAGAG | | pCPC | CPC-F2-sen-attB1 | GGGGACAAGTTTGTACAAAAAAGCAGGCTATCGAATTCCTGAACTTTATATCACAGTCC | |  | CPC-F2-asen-attB2 | GGGGACCACTTTGTACAAGAAAGCTGGGTGGCTCGAAAAGGAAAAGAAAATCAAAC | | A3,B mutMYB1 | MP-TRY-mut-MYB1-for | ggtacgggggcccctgtatgtccaacc | |  | MP-TRY-mut-MYB1-rev | ggttggacatacaggggcccccgtacc | | A3,B mutMYB2 | MP-TRY-mut-MYB2-for | ggtgtgtatgtgggcccctgaaaaaaaaac | |  | MP-TRY-mut-MYB2-rev | gtttttttttcaggggcccacatacacacc | | A3,B mutMYC1 | MP-TRY-mut-MYC1-for | cgttgaacttggggcccccaaataaaaatc | |  | MP-TRY-mut-MYC1-rev | gatttttatttgggggccccaagttcaacg | | A3,B mutMYC2 | MP-Pr23-mutMYC-Fr2-for | CAATAAAAGACTTCATCTCCCCGGGTATTTGAGTG | |  | MP-Pr23-mutMYC-Fr1-rev | CACTCAAATACCCGGGGAGATGAAGTCTTTTATTG | |
| --- | --- | --- | --- | --- | --- | --- | --- | --- | --- | --- | --- | --- | --- | --- | --- | --- | --- | --- | --- | --- | --- | --- | --- | --- | --- | --- | --- | --- | --- | --- | --- | --- | --- | --- | --- | --- | --- | --- | --- | --- | --- | --- | --- | --- | --- | --- | --- | --- | --- | --- | --- | --- | --- | --- | --- | --- | --- | --- | --- | --- | --- | --- | --- | --- | --- | --- | --- | --- | --- | --- | --- | --- | --- | --- | --- | --- | --- | --- | --- | --- | --- | --- | --- | --- | --- | --- | --- |
